# The effectiveness of intratympanic injections with methylPREDnisolon versus placebo in the treatment of vertigo attacks in MENière’s disease (PREDMEN trial): a study protocol for a phase-3 multicentre, double-blinded, randomised, placebo-controlled trial

**DOI:** 10.1136/bmjopen-2023-076872

**Published:** 2024-08-29

**Authors:** Maud Martina Emilie Boreel, Babette van Esch, Tjard R Schermer, Berber M Mol, Peter Paul van Benthem, Tjasse D Bruintjes

**Affiliations:** 1 Department of Otorhinolaryngology—Head and Neck Surgery, Leiden University Medical Center, Leiden, Zuid-Holland, The Netherlands; 2 Apeldoorn Dizziness Centre, Gelre Ziekenhuizen, Apeldoorn, Gelderland, The Netherlands

**Keywords:** otolaryngology, adult otolaryngology, randomized controlled trial

## Abstract

**Introduction:**

Intratympanic corticosteroids are commonly used in the treatment of Menière’s disease (MD). However, few and small randomised controlled trials (RCT) on the effectiveness of intratympanic corticosteroids have been performed. A recent Cochrane review suggested that a well-conducted placebo-controlled RCT with a large study population is required to evaluate the effectiveness of the use of intratympanic corticosteroids in MD. The following protocol describes a phase-3 multicentre, double-blinded, randomised, placebo-controlled trial to compare the effectiveness of methylprednisolone (62.5 mg/mL) to a placebo (sodium chloride 0.9%).

**Methods and analysis:**

We aim to recruit 148 patients with unilateral MD from six hospitals in the Netherlands. Patients will be randomly assigned to either the methylprednisolone or the placebo group. Two injections will be given, one at baseline and one after 2 weeks. Follow-up assessments will be done at 3, 6, 9 and 12 months. The primary outcome will be the frequency of vertigo attacks. Attacks will be evaluated daily with the DizzyQuest app. Secondary outcomes include hearing loss, tinnitus, health-related quality of life, use of co-interventions and escape medication, (serious) adverse events and cost-effectiveness. These will be evaluated with audiometry and multiple commonly used, validated questionnaires. For the primary and secondary outcomes mixed model analysis, generalised estimating equation analysis and logistic regression analysis will be used.

**Ethics and dissemination:**

This study was submitted via the Clinical Trials Information System, reviewed and approved by the Medical Research Ethics Committee Leiden The Hague Delft and the local institutional review board of each participating centre. All data will be presented ensuring the integrity and anonymity of patients. Results will be published in scientific journals and presented on (inter)national conferences.

**Trial registration number:**

This study is registered at ClinicalTrials.gov Protocol Registration and Results System, with the registration ID: NCT05851508.

**STRENGTHS AND LIMITATIONS OF THIS STUDY**

- In this randomised placebo-controlled study, both participants and clinicians will remain blinded throughout the follow-up period, therefore, minimising the risk of bias.
- The prospective design with patients daily recording their vertigo attack directly in an app lowers the risk of missing data and recall bias.
- This study includes a rather large patient population of 148 patients.
- Vestibular migraine and Menière’s disease (MD) share multiple features in terms of clinical presentation and other symptomatology, distinguishing between the two could be challenging and therefore, could form a possible limitation in this study.
- Subanalyses on clinical subgroups of MD (autoimmune, familial and MD+migraine) will be difficult to conduct because many patients cannot be classified in a subgroup or are part of multiple subgroups.

## Introduction

 Menière’s disease (MD) is a clinical condition characterised by tinnitus and aural fullness, low-frequency to mid-frequency sensorineural hearing loss, and spontaneous episodes of vertigo that can last 20 min to 12 hours.^1^ Patients with MD experience a worse quality of life than healthy patients due to vertigo, tinnitus and hearing loss.^2^ In addition, higher levels of anxiety and depression are seen in patients with MD.^3^

Although its aetiology is unknown, endolymphatic hydrops (EH) is thought to be associated with MD. Almost all patients with MD have EH, but not all patients with EH have symptoms of MD. It is unknown if EH is a result of MD or a causal factor for MD.^4 5^ Until this day, there is no agreement as to the ideal treatment of MD due to the lack of evidence for the effect of various therapies. Current treatment consists of dietary and lifestyle modifications, oral diuretics, vestibular rehabilitation for chronic imbalance, intratympanic therapy and/or ablative surgery.^1^ With intratympanic gentamicin and corticosteroid injections, the drug is directly delivered into the middle ear, from where it will be absorbed in the inner ear. Unlike gentamicin, corticosteroid therapy does not carry a risk of causing hearing loss. Therefore, it is currently the first step of standard care in the treatment of MD.^1^ Although the mechanism of action of steroids on the inner ear remains speculative, it may improve cochlear blood flow and stabilise the vascular endothelium which enhances fluid homeostasis by upregulation of cochlear ion gene expression.^6^ Recently, a Cochrane review was published evaluating the use of intratympanic corticosteroids in MD.^7^ In this review, 10 randomised controlled trials (RCTs) and quasi-RCTs comparing intratympanic corticosteroids, all using dexamethasone, compared with either placebo or no treatment were included. The authors found that the evidence for the use of dexamethasone is uncertain. Intratympanic dexamethasone injection may marginally reduce the frequency of vertigo attacks. Regarding hearing and tinnitus, improvement was seen but without statistical significance.

The most commonly intratympanically administered corticosteroids are dexamethasone and methylprednisolone.^1^ Phillips *et al*^8^ determined the efficacy of intratympanic OTO-104 (a sustained-released dexamethasone hydrogel) for the treatment of MD, in three double-blind, placebo-controlled RCTs, with a total of 165, 174 and 148 patients respectively. OTO-104 showed numerically larger decreases in definitive vertigo days compared with placebo across all three studies. However, in only one study, this difference was statistically significant. Pharmacokinetic studies show that dexamethasone phosphate has molecular and pharmacokinetic characteristics that complicate its use as a topical therapy for hearing disorders, which may explain its questionable effectiveness.^9^ An animal study found that the concentrations of methylprednisolone are higher and have longer duration in perilymph and endolymph compared with dexamethasone and hydrocortisone, and therefore, could be a more effective drug.^10^ Typically, soluble forms of methylprednisolone are administered and expected to be less permeable through the membranous boundaries compared with the less polar forms. However, there is no data whether these soluble forms are metabolised to the base form in the ear and if they are, at what rate.^9^ Despite the fact that little is known about the pharmacokinetics of methylprednisolone, there are clinical indications of its effectiveness.^11 12^ Cao *et al*^12^ performed a literature review and demonstrated that methylprednisolone is more effective than dexamethasone in a clinical setting.

Although in the last decade, there is an increasing tendency and emerging evidence for the use of intratympanic steroids, no large RCT on the effectiveness of intratympanic methylprednisolone in MD has been conducted.^13^ A meta-analysis published in 2021 included eight studies comparing intratympanic gentamicin to intratympanic corticosteroids, in which gentamicin appeared to be superior in terms of control of vertigo attacks.^14^ However, gentamicin is known to be ototoxic and can induce hearing loss. Patel *et al*^15^ compared intratympanic gentamicin injections to methylprednisolone injections in a double-blind RCT with a 24-month post-treatment follow-up. Vertigo attacks decreased in both groups, indicating a treatment effect. However, no placebo group was involved and the sample size was relatively small (n=60).

In conclusion, there is a need of solid evidence on the effectiveness of intratympanic steroids in MD. Until now, the effectiveness of methylprednisolone has not been investigated by means of a placebo controlled RCT. Therefore, a well-conducted RCT with a large study population and a long follow-up period is now required to evaluate the effectiveness of intratympanic methylprednisolone in MD. In this protocol, we present the methods of a phase-3 multicentre, double-blinded, randomised, placebo-controlled trial evaluating the effectiveness of intratympanic injections with methylprednisolone vs placebo in the treatment for MD patients.

## Methods

### Trial design

In this phase 3, multicentre, double-blind, placebo-controlled randomised trial, the effect of two intratympanic methylprednisolone sodium succinate 62.5 mg/mL (Solu-Medrol in Act-O-Vial, Pfizer BV) injections 14 days apart is compared with two placebo (ie, sodium-chloride 0.9%) injections with the same time interval on vertigo attacks in patients with MD. Parallel groups will be randomly assigned to one of both arms and outcomes will be measured during a 1 year follow-up period.

To ensure transparency and completeness in this clinical trial, the SPIRIT and CONSORT checklists are provided in onlinesupplemental files 1  2.

### Study subjects

Patients with MD will be recruited by six participating sites in the Netherlands and will be approached by their own ENT specialist and informed about this trial. After a 1 week reflection period, informed consent forms can be signed. Baseline and outcome data will be extracted from participants’ electronic medical records and collected in the cloud-based clinical data management platform Castor EDC (version v2023.1.0.1, LUMC).

### In- and exclusion criteria

In order to be eligible for the study, a study subject needs to have unilateral, definite MD according to the diagnostic criteria derived from the American Academy Otolaryngology Head and Neck Surgery, Classification Committee of the Bárány Society, European Academy of Otology and Neurotology and International Classification of Vestibular Disorders published in 2015.^16^ The criteria for definite MD are:

Two or more spontaneous episodes of vertigo, each lasting 20 min to 12 hours,

AND

Audiometrically documented low-frequency to medium-frequency sensorineural hearing loss in one ear, defining the affected ear on at least one occasion before, during or after one of the episodes of vertigo,

AND

Fluctuating aural symptoms (hearing, tinnitus or fullness) in the affected ear (not better accounted for by another vestibular diagnosis).

Other inclusion criteria are:

- Age >18 years at the start of the trial.
- ≥ 4 vertigo attacks over the last 6 months.
- Willing to adhere to daily completion of study questionnaires using the DizzyQuest app and to the follow-up assessments.

Study subjects who meet any of the following criteria will be excluded:

- Bilateral MD.
- Severe disability (eg, neurological, orthopaedic, cardiovascular) or serious concurrent illness that might interfere with treatment or follow-up.
- Active additional neuro-otologic disorders that may mimic MD (eg, vestibular migraine, recurrent vestibulopathy, phobic postural vertigo, vertebro-basilar TIAs, acoustic neuroma).
- Otitis media with effusion based on tympanogram results.
- History of intratympanic injections with corticosteroid less than 6 months ago.
- History of intratympanic injections with gentamicin or ear surgery for treating MD.
- Pregnant women or nursing women.

### Sample size

A sample size calculation was performed based on recommendations as summarised in the Clinical Practice Guideline for Menière’s disease.^1^ An expected proportion of subjects achieving vertigo control of 87.5% was assumed for methylprednisolone treatment compared with an assumed 67.5% for placebo, that is, a difference in treatment effect of 20%. With a statistical power (1-β) of 80% and a type 1 error (α) of 5%, 67 patients per group are required. With an estimated 10% loss-to-follow-up, 74 patients will be included in each arm, giving a total sample size of 148. In total, over the six participating centres, 340 MD patients yearly visit the Otolaryngology department and will be screened for the trial. It is expected that 15% will meet the inclusion criteria and will be willing to participate. This will result in approximately 50 eligible patients for inclusion per year.

### Randomisation and blinding

Subjects will be randomly assigned to either methylprednisolone or placebo with a 1:1 allocation as per computer-generated random sequence, stratified by site generated by Castor EDC. Blinding will be maintained until all subjects have finished their treatment phases. All study participants, participating medical professionals and outcome assessors will be blinded. The independent epidemiologist and pharmacy personnel will both be unblinded during randomisation and therapy allocation.

### Study procedure

After a 1 week reflection period and agreement with trial participation by means of signing the informed consent, a patient will be seen at the study site. Standard inquiries about the patient’s demographics, family history and medical history—particularly regarding any history of autoimmune disease and migraine—are made at the informed consent visit. Thereafter, patients will receive an intratympanic injection with either methylprednisolone or placebo at day 1 and day 15 with a window of 3 days. The patient is lying down in supine position with their head rotated to the side and prior to the intratympanic injection, the eardrum will be anaesthetised. Thereafter, a myringotomy is being performed and a small spinal puncture needle is passed through the tympanic membrane to inject fluid into the middle ear cavity at the level of the round window. The patient is then required to remain on their side without swallowing for 30 min.

At baseline, results of MRI must be available to make sure other causes of disease are ruled out.^17^ To assess the vestibular function of the horizontal semicircular canals, the caloric test and video head impulse test (vHIT) will be performed. To evaluate the anterior and posterior semicircular canals, the vHIT will be conducted at baseline in order to assess the presence of vestibular hypofunction or areflexia.

**Figure 1:**
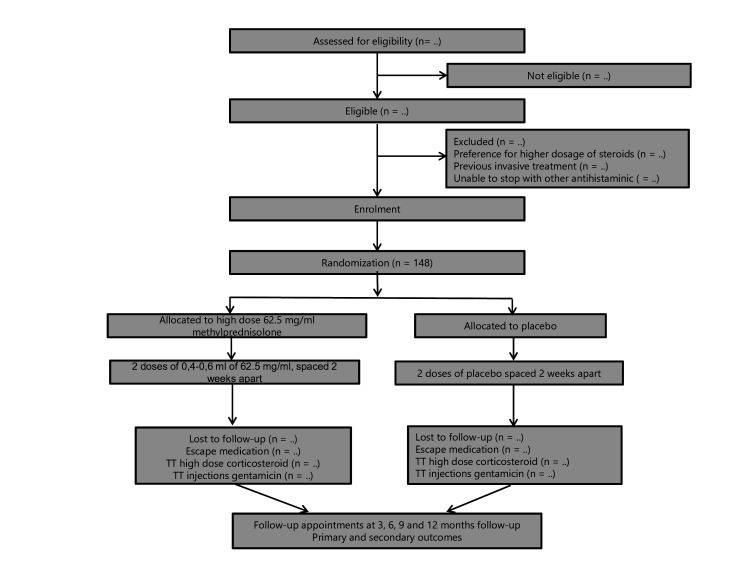
Flowchart of the study procedure. IT, intratympanic.

During a follow-up period of 1 year, overall well-being and vertigo attacks are being assessed daily with the aid of the DizzyQuest app. Two telephone contacts will take place at 3 and 6 months to assess possible (serious) adverse events, DizzyQuest app compliance and the use of escape medication or co-interventions. A physical follow-up visit in the outpatient clinic will be scheduled at 6 and 12 months. During these outpatient clinic visits, additional audiometry and multiple questionnaires concerning tinnitus, dizziness and quality of life will be filled-in. An overview of follow-up moments of the corresponding outcomes and a flowchart of the study procedure are presented in table 1 and figure 1, respectively.

**Table 1:** Overview of follow-up moments of the corresponding outcomes

Moment in trial	Type of follow-up	Outcomes
From moment of inclusion unit 1 year follow-up		Daily questionnaire in DizzyQuest app
Baseline	First intratympanic injection	Baseline parametersMRIOtoscopyAudiometry PTA and SDS Vestibular testing vHIT, caloric testing Questionnaires DHI, TFI, FLS, EQ-5D/VAS, iMCQ, iPCQ
2 weeks	Second intratympanic injection	OtoscopyComplicationsUse of co-interventionsUse of escape medication(S)AE
3 months, 9 months	Telephone consult	ComplicationsUse of co-interventionsUse of escape medication(S)AE
6 months, 12 months	Consult in outpatient clinic	OtoscopyComplicationsAudiometry PTA and SDS Questionnaires DHI, TFI, FLS, EQ-5D/VAS, iMCQ, iPCQ Use of co-interventions Use of escape medication (S)AE

DHI, Dizziness Handicap Index; EQ 5DEuroQol 5DFLS, Functional Level Scale; HIT, head impulse test; iMCQ, iMTA Medical Consumption Questionnaire; iPCQ, iMTA Productivity Cost; PTA, pure tone average; (S)AE, (Serious) Adverse EventSDS, Speech Discrimination ScoreTFITinnitus Functional Index

### Outcome measures

#### Vertigo

The primary outcome measure will be defined by the class of vertigo as defined by the AAO HNS 1995 guideline. The class of vertigo is defined by the average number of attacks per month during 6 and 12 months after treatment divided by the number of attacks 6 months before treatment times 100. As a result, the following class of vertigo is defined:

- A: Complete control of vertigo=0.
- B: Substantial control of vertigo=1–40.
- C: Limited control of vertigo=41–80.
- D: Insignificant control of vertigo=81–120’.
- E: Worse control of vertigo >120.

Moreover, the daily attack vertigo frequency will be monitored with the aid of the DizzyQuest app (Psymate 2).^18 19^ The DizzyQuest app will be used to track the primary outcome measure, the frequency of dizziness attacks. Patients will answer daily questionnaires about their health and well-being and patients can report vertigo attacks at any time using the DizzyQuest app. Additionally, the Dizziness Handicap Inventory (DHI) questionnaire will be administered at baseline, after 6 months and after a year to assess how dizziness affects daily life.^20^ The effect of the injections in the DHI will be reported as raw data, as well as change in handicap category (mild handicap, moderate handicap or severe handicap) related to improvement, unchanged or worsened.

The following secondary outcomes will be measured.

#### Hearing

Pure tone audiometry will be performed at baseline, after 6 months and after a year. In line with the guideline of AAO HNS 1995 guideline,^17^ we will use the average scores of four-tone audiometry at 0.5, 1, 2 and 3 kHz and we will assess the word recognition scores as the percent correct score at the presentation level in decibel. A decrease of ≥10 dB or a change in word recognition score of ≥15% points is considered clinically significant.

#### Tinnitus

The Tinnitus Functional Index (TFI) measures the impact of tinnitus on daily life.^21^ This survey will be administered at baseline, after 6 months and after a year. One point decrease of increase will be defined as improved or worsened tinnitus respectively.^22^

#### Quality of life

Apart from the DHI and TFI, the EuroQol 5 dimension (EQ-5D) and EuroQol – Visual Analogue Scale (EQ-VAS) questionnaires will be used to measure quality of life at baseline, after 6 and 12 months.^23 24^ These questionnaires are standardised tests of health status that are used in economic and clinical evaluations.

#### Use of escape medication and co-interventions

In case participants remain suffering from intolerable vertigo attacks, regardless of which group they are allocated to, the use of metoclopramide and co-interventions such as intratympanic injections of gentamicin or methylprednisolone will be allowed and documented during the follow-up period. This will be based on experience of participants’ vertigo frequency and shared decision making. If patients receive additional treatment, they will not be unblinded.

#### Adverse events

Patients will be informed that Adverse Events, Serious Adverse Events and Suspected Unexpected Serious Adverse Reactions must be reported as soon as possible to their ENT-surgeon or research nurse. Additional queries are made at 3, 6, 9 and 12 months to ensure that they did not fail to report occurrences. These events will be registered throughout the trial in Castor EDC. Each serious adverse event must be reported to the sponsor within 24 hours after the physicians’ knowledge. In the event that patient safety is compromised, patients can be unblinded.

#### Cost-effectiveness

Cost-effectiveness will be assessed from both a healthcare and societal cost-utility perspective, where cost per avoided vertigo attack and cost per Quality Adjusted Life Year (QALY), respectively, will be used as the metrics. MD-related medical expenses, other healthcare expenses and the cost of lost productivity will all be included in the estimated societal cost, which will be calculated using the iMTA Medical Consumption Questionnaire (iMCQ) and IMTA Productivity Cost Questionnaire (IPCQ).

### Statistical analysis

Ordinal regression using mixed model analysis will be used to analyse the primary outcome (ie, class of vertigo). In addition, generalised estimating equation analysis of the actual vertigo attacks recorded using the DizzyQuest app will be used to estimate the incidence rate ratio for comparison between the methylprednisolone and placebo groups. A decrease of 100% is considered total control of vertigo episodes, while a reduction of >40% is considered a substantial and thus clinically relevant reduction.^17^

Mixed model analysis will be used to analyse differences in the questionnaire scores (DHI, TFI, FLS, eQ-5D/VAS, iMCQ, iPCQ) between the two groups. Logistic regression analysis will be used to analyse the remaining secondary outcomes (incidence of escape interventions, hearing loss and adverse events). A reduction in hearing of 10 decibels or a 15% change in word recognition will be regarded as a clinically significant difference.^17^

Subgroup analyses will be performed with regard to sex, duration of the disease and the type of MD (delayed MD, familial MD and autoimmune MD). These subgroups will be defined as described in Frejo *et al*.^25^ Two sensitivity analyses will be carried out in addition to the intention to treat analysis: a per protocol analysis in which patients who received additional co-interventions to achieve vertigo control are excluded; and an as-treated analysis in which participants who received additional co-interventions are analysed.

In order to evaluate the average costs and outcomes between the methylprednisolone and placebo groups for the cost-effectiveness analysis, intention-to-treat and net-benefit analysis will be used. For all statistical analysis, multiple imputation to adjust for missing data will all be used.^26^ QALYs will be calculated using the Dutch tariff for the EuroQoL EQ-5D-5L^23^ and as sensitivity analysis, the visual analogue scale valuing health, with power-transformation.^27^ All outcomes with corresponding statistical analysis methods are summarised in table 2. A p value <0.05 will be considered as statistically significant for all analyses and will be performed using SPSS V.25 or higher (SPSS Chicago Illinois, USA).

**Table 2:** Outcomes with corresponding statistical analysis method

	Outcome	Type of data	Analysis
Primary outcome	Number of spontaneous vertigo attacks, lasting more than 20 min	Count	Generalised estimating equation
	AAO HNS (1995) class of vertigo		Mixed model analysis
Secondary outcomes	Hearing (PTA; 0.5, 1, 2, 3 kHz and SDS)	Continuous	Logistic regression analysis
	Tinnitus (TFI)	Categorical	Mixed model analysis
	Quality of life (DHI, TFI, FLS, eQ-5D/VAS)	Categorical	Mixed model analysis
	Use of escape medication and co-interventions	Count	Logistic regression analysis
	(S)AE	Binary	Logistic regression analysis
	Cost effectiveness (iMCQ, iPCQ)	Categorical	Mixed model analysis

DHI, Dizziness Handicap Index; EQ 5DEuroQol 5DFLS, Functional Level Scale; iMCQ, iMTA Medical Consumption Questionnaire; iPCQ, iMTA Productivity Cost; PTA, pure tone average; (S)AE, (Serious) Adverse EventSDS, Speech Discrimination ScoreTFITinnitus Functional Index

### Patient and public involvement

The PREDMEN trial is supported by the Dutch Association for the hard of hearing, and more specifically, its Committee Dizziness and Balance (Commissie Duizeligheid en Evenwicht van Hoormij-NVVS) and the Dutch association for psychological healthcare and social services for patients with SNHL and tinnitus (GGMD). Both organisations are involved in the realisation of the trial, the writing process and implementation of trial results. Moreover, they will serve as a sounding board for MD patients participating in this trial and one patient representative will be a member of the steering committee. In line with their suggestions, patients will be involved in every stage of the research.

## Ethics and dissemination

### Ethics

The PREDMEN trial was submitted via the Clinical Trial Information System (CTIS), with CTIS number: *2023-503340-13-00*, reviewed by the Medical Research Ethics Committee Leiden The Hague Delft (MREC LDD), and authorised for execution in the Netherlands under the European Clinical Trial Regulation (ECTR). Additionally, the institutional research board and the Board of Directors of each participating centre (Franciscus Gasthuis & Vlietland, Gelre ziekenhuizen, HagaZiekenhuis, Leiden University Medical Centre, Maastricht University Medical Centre, Medisch Spectrum Twente) individually reviewed and approved the study. The study is conducted in accordance with the principles outlined in the Declaration of Helsinki (October 2013), the Medical Research Involving Human Subjects Act (WMO, 26 February 1998), the International Conference on Harmonisation Good Clinical Practice (ICH GCP, November 2016) guidelines, and any other applicable guidelines, regulations and Acts.

### Patient safety

Minor complications such as persistent membrane perforation (5.9%) and otitis media (7%) can occur.^7 28 29^ Safety risk will be comparable to normal clinical practice and it is not expected that significant adverse events will be seen in the intervention arm. ENT specialists are experienced with intratympanic injections due to its application in patients with sudden deafness. Since the intervention is characterised as a low risk profile study, no Data Safety Monitoring Board (DSMB) is required.^30^ The sponsor will submit a report on the safety of each investigational medicinal product used in the clinical trial through CTIS. Interim analysis will be performed on the primary endpoint when 50% of the patients have been randomised and completed a follow-up of 6 months, where comparability of baseline characteristics will be assessed. In these analyses, differences in vertigo control between the two study arms should not be greater than 45%. In addition, if the difference in vertigo control reveals to be clinically significant (ie, >20%), but ≤20% of the participants in methylprednisolone reach vertigo control, the study will be terminated because of convincing effect of the treatment in the intervention arm.

### Data safety

The handling of personal data complies with the Dutch Personal Data Protection Act (AVG). All data collected for the trial, including but not limited to demographic data, audiological questionnaires and data from the DizzyQuest app will be entered in a ISO 9001 and ISO 27001:2005 certified Castor EDC database (electronic CRF). Data will be protected with a unique subject identification code which is linked to a password protected subject identification list. Only members of the study team, who will be documented on the site signature and delegation log per site, will have access to the study data. The sponsor and investigator will keep a clinical trial master file which will contain the essential documents relating to the clinical trial.

### Dissemination

A summary of the results of this study will be submitted to CTIS within 1 year after termination of the trial. Results will also be published in scientific journals and presented on (inter)national conferences. All information that will be presented will be done so in a way that integrity and anonymity of patients are ensured. All data will be stored for 25 years after the last subject has had the last study visit.

### Trial registration

This study is registered at ClinicalTrials.gov Protocol Registration and Results System, with the registration ID: NCT05851508.

## supplementary material

10.1136/bmjopen-2023-076872 online supplemental file 1

10.1136/bmjopen-2023-076872 online supplemental file 2
