# Supplementary material for: The effectiveness of intratympanic injections with methylPREDnisolon versus placebo in the treatment of vertigo attacks in MENière’s disease (PREDMEN trial): a study protocol for a phase-3 multicentre, double-blinded, randomised, placebo-controlled trial
Source: BMJ Open. 2024 Aug 29;14(8):e076872. doi: 10.1136/bmjopen-2023-076872 (PMC11367374; doi:10.1136/bmjopen-2023-076872)
Supplement: online supplemental file 2 [file bmjopen-14-8-s002.pdf]

SPIRIT

STANDARD PROTOCOL ITEMS: RECOMMENDATIONS FOR INTERVENTIONAL TRIALS

SPIRIT 2013 Checklist: Recommended items to address in a clinical trial protocol and related documents*

Section/item	Item No	Description
**Administrative information**		
Title	1	**Descriptive title identifying the study design, population, interventions, and, if applicable, trial acronym**<br>A phase-3 multicentre, double-blinded, randomised, placebo-controlled trial to compare the effectiveness of intratympanic injections with methylPREDnisolon versus placebo in the treatment of vertigo attacks in MENière's disease (PREDMEN trial).
Trial registration	2a	**Trial identifier and registry name. If not yet registered, name of intended registry**<br>Clinical trial number: 2023-503340-13-00<br>Clinicaltrails.gov: ID: NCT05851508
	2b	**All items from the World Health Organization Trial Registration Data Set**

Data category	Information
Primary registry and trial identifying number	ClinicalTrials.gov NCT05851508
Date of registration in primary registry	3, oktober, 2023
Secondary identifying numbers	Identifiers: NCT05851508, Unique Protocol ID: PREDMEN, Secondary ID: 10140022110009
Source(s) of monetary or material support	Stichting ZonMW: *file number: 10140022110009*
Primary sponsor	Leiden University Medical Centre
Secondary sponsor(s)	Leiden University Medical Centre
Contact for public queries	M.M.E. Boreel, MD, Leiden University Medical Centre M.M.E.Boreel@lumc.nl
Contact for scientific queries	M.M.E. Boreel, MD, Leiden University Medical Centre M.M.E.Boreel@lumc.nl
Public title	The Effectiveness of Intratympanic Methylprednisolone Injections Compared to Placebo in the Treatment of Vertigo Attacks in Meniere's Disease (PREDME
Scientific title	A Multicenter, Double-blinded, Randomised, Placebo-controlled Trial to Compare the Effectiveness of Intratympanic Injections

	MethylPREDnisolone Versus Placebo in the Treatment of Vertigo Attacks in MENière's Disease (PREDMEN Trial).
Countries of recruitment	Netherlands
Health condition(s) or problem(s) studied	Meniere's Disease (MD)
Intervention(s)	Intratympanic injection with Methylprednisolone 62.5 mg/ ml
	Placebo comparator: Intratympanic injection with saline, (sodiumchloride 0.9%)
Key inclusion and exclusion criteria	
	Inclusion criteria:<br>- Unilateral, definite MD according to the diagnostic criteria derived from the American Academy Otolaryngology Head and Neck Surgery, Classification-Page 4 of 6 Committee of the Bárány Society, European Academy of Otology and Neurotology and International Classification of Vestibular Disorders<br>- adult patients (≥ 18 years), patient hospitalized<br>- ≥ 4 vertigo attacks over the last 6 months.<br>- Willing to adhere to daily Dizzy quest app questionnaires and the follow-up assessments
	Exclusion criteria:<br>- Bilateral MD<br>- Severe disability (e.g. neurological, orthopaedic, cardiovascular) or serious concurrent illness that might interfere with treatment or follow-up.<br>- Active additional neuro-otologic disorders that may mimic MD (e.g. vestibular migraine, recurrent vestibulopathy, phobic postural vertigo, vertebro-basilar TIAs, acoustic neuroma).<br>- Otitis media with effusion based on tympanogram results.<br>- History of intratympanic injections with corticosteroid less than 6 months ago.<br>- History of intratympanic injections with gentamicin or ear surgery for treating MD.<br>- Pregnant women and nursing women.
Study type	Interventional
	Allocation: randomised intervention model.<br>Masking: double blind (subject, caregiver, investigator, outcomes assessor)
	Primary purpose: treatment
	Phase III
Date of first enrolment	Oktober 2023
Target sample size	148
Recruitment status	Recruiting
Primary outcome(s)	Vertigo attacks
Key secondary outcomes	Hearing loss, tinnitus, health-related quality of life, use of escape medication, adverse events, cost-effectiveness, co-interventions, overall function, impact of dizziness.

Protocol version 3 **Date and version identifier**

- Issue date: 02-05-2023
- Version 4.0

Funding	4	**Sources and types of financial, material, and other support**
		This project will be funded by ZonMW *file number: 10140022110009*

Roles and responsibilities — 5a — **Names, affiliations, and roles of protocol contributors**

Coordinating investigators:

- T.D. Bruintjes, MD, PhD, prof, ENT-surgeon, department of Otorhinolaryngology Head and Neck surgery.
- M.M.E. Boreel, MD, physician-researcher, department of Otorhinolaryngology Head and Neck surgery.

Head of research support Leiden University Medical Centre:

- B.M. Mol, PhD, Head of research support Leiden University Medical Centre

Principal investigators:

- B.F. van Esch, MD, PhD, ENT surgeon, Department Otorhinolaryngology and Head and Neck Surgery, Leiden University Medical Centre, The Netherlands
- S.M. Winters, MD, PhD , ENT-surgeon Apeldoorn Dizziness Centre, Gelre Ziekenhuizen
- W.P.A. Kelders, MD, PhD , ENT-surgeon , Rotterdam Dizziness Centre, Franciscus Gasthuis & Vlietland
- R. van den Berg, MD, PhD, ENT-surgeon Department of Otorhinolaryngology, Maastricht UMC+
- H.M. Blom, MD, PhD, Professor, ENT-surgeon Department of Otorhinolaryngology, Haga Hospital
- D.A. van Zuylen, MD, ENT-surgeon, Department of Otorhinolaryngology, Medisch Spectrum Twente

Epidemiologist:

- T. Schermer, PhD, Apeldoorn Dizziness Centre, Gelre Hospital, Apeldoorn, The Netherlands

5b — **Name and contact information for the trial sponsor**

Legal Representative of Sponsor: mw. K.G. Freriks-Bauritius Title: Management director division 3. Leiden University Medical Centre, Albinusdreef 2, 2333 ZA Leiden

secretariaatbbd3enzorg@lumc.nl.

5c — **Role of study sponsor and funders, if any, in study design; collection, management, analysis, and interpretation of data; writing of the report; and the decision to submit the report for publication, including whether they will have ultimate authority over any of these activities**

The funding source does not have any role in this study during its execution, analysis, interpretation of the data or decision to submit results.

5d **Composition, roles, and responsibilities of the coordinating centre, steering committee, endpoint adjudication committee, data management team, and other individuals or groups overseeing the trial, if applicable (see Item 21a for data monitoring committee)**

Coordinating investigator:

- Study planning
- Randomisation
- Responsible for trial master file
- Planning of monitor visits
- Identification Study subjects, recruitment, data collection and completion of CRFs, along with follow up of study patients and adherence to study protocol and investigators brochure
- Reporting of AE/SAE/SUSAR
- Processing and analysing raw data

Coordinating investigator/PI sponsor site/head of research support LUMC:

- Design and Conduct of the PREDMEN trial
- Preparation, writing and revisions of research protocol, agreement on final protocol
- Write and review all required documents (Patient information folder, Informed Consent, Standard operating procedures etc.)
- Organisation of approval medical ethical committee
- Preparation of Investigators brochure and CRF
- Publication study reports
- Reviewing progress of study and if necessary, making changes to the protocol or other necessary documents
- Management of the budget and problems with individual centrecontracts
- Provide annual risk report

Principal investigators/Research nurse Study sites:

- Identification Study subjects, recruitment, data collection and completion of CRFs, along with follow up of study patients and adherence to study protocol and investigators brochure

Epidemiologist:

- Performing interim analysis
- Processing and analysing raw data

Introduction

Background and rationale

6a **Description of research question and justification for undertaking the trial, including summary of relevant studies (published and unpublished) examining benefits and harms for each intervention**

Menière's disease (MD) is a clinical condition characterized by tinnitus and aural fullness, low- to mid-frequency sensorineural hearing loss, and spontaneous episodes of vertigo that can last 20 minutes to 12 hours [1]. Although its aetiology is unknown, endolymphatic hydrops (EH) is thought to be associated with MD and treatment is often focussed on the regulation of EH.

Current treatment of MD consists of dietary and lifestyle modifications, oral diuretics, vestibular rehabilitation for chronic imbalance, intratympanic therapy, and/or ablative surgery [1]. With intratympanic gentamicin and intratympanic corticosteroid injections the drug is directly delivered into the middle ear, from where it will be absorbed in the inner ear. Although the mechanism of action

of steroids on the inner ear remains speculative it may improve cochlear blood flow and stabilize the vascular endothelium which enhances fluid homeostasis by upregulation of cochlear ion gene expression [2]. Unlike gentamicin, corticosteroid therapy does not carry a risk of causing hearing loss. Therefore, it is currently the first step of standard care in the treatment of MD [1].

Although in the last decade, there has been an increasing tendency and emerging evidence for the use of intratympanic steroids, no large RCT on the effectiveness of intratympanic methylprednisolone in MD has been conducted. A meta-analysis published in 2021 included eight studies comparing intratympanic gentamicin to intratympanic corticosteroids, in which gentamicin appeared to be superior in terms of control of vertigo attacks [3]. However, gentamicin is known to be ototoxic and can induce hearing loss. Patel et al. compared intratympanic gentamicin injections to methylprednisolone injections in a double-blind RCT with a 24-month post-treatment follow-up [4]. Vertigo attacks decreased in both groups, indicating a treatment effect. However, no placebo group was involved and the sample size was relatively small (n=60). Recently, a Cochrane review was published evaluating the use of intratympanic corticosteroids in MD. In this review, 10 randomised controlled trials (RCTs) and quasi-RCTs comparing intratympanic corticosteroids, all using dexamethasone, compared to either placebo or no treatment were included [5]. The authors found that the evidence for the use of dexamethasone is uncertain. Intratympanic dexamethasone injection may marginally reduce the frequency of vertigo attacks. Regarding hearing and tinnitus improvement was seen, but without statistical significance.

In conclusion, there is a need of solid evidence on the effectiveness of intratympanic steroids in MD. Until now the effectiveness of methylprednisolone has not been investigated by means of a placebo controlled RCT. Therefore a well-conducted RCT with a large study population and a long follow-up period is now required to evaluate the effectiveness of intratympanic methylprednisolone in MD.

6b **Explanation for choice of comparators**

Pharmacokinetic studies show that dexamethasone phosphate has molecular and pharmacokinetic characteristics that complicate its use as a topical therapy for hearing disorders, which may explain its questionable effectiveness [6]. An animal study found that the concentrations of methylprednisolone are higher and have longer duration in perilymph and endolymph compared to dexamethasone and hydrocortisone, and therefore could be a more effective drug [7]. Typically soluble forms of methylprednisolone are administered and expected to be less permeable through the membranous boundaries compared to the less polar forms. However, there is no data whether these soluble forms are metabolized to the base form in the ear and if they are, at what rate. Despite the fact that little is known about the pharmacokinetics of methylprednisolone, there are clinical indications of its effectiveness [8, 9]. Cao et al. performed a literature review and demonstrated that methylprednisolone is more effective than dexamethasone in a clinical setting [8]. Therefore we choose to compare methylprednisolone to placebo.

Objectives 7 **Specific objectives or hypotheses**

Primary objective: To determine whether methylprednisolone (62.5 mg/ml) is superior to placebo in controlling vertigo attacks in patients with MD.

Secondary objective: To determine whether methylprednisolone (62.5 mg/ml) is superior to placebo in controlling tinnitus, hearing, quality of life, use of escape medication, adverse events and cost-effectiveness.

Trial design 8 **Description of trial design including type of trial (eg, parallel group, crossover, factorial, single group), allocation ratio, and framework (eg, superiority, equivalence, noninferiority, exploratory)**

The PREDMEN study is a phase-3 multicentre, double-blinded, randomized, parallel group trial with patients suffering from MD with a total follow-up period of 12 months.

Methods: Participants, interventions, and outcomes

Study setting	9	**Description of study settings (eg, community clinic, academic hospital) and list of countries where data will be collected. Reference to where list of study sites can be obtained**

Six medical centres from across the Netherlands are participating in the study in order to enrol a sufficient number of patients in three years. In these centres, an ENT-surgeon, specialized in dizziness, is involved and principal investigator for the trial. The following centres are participating in the PREDMEN study:

- Leiden Universitair Medisch Centrum, Leiden (sponsor site)
- Apeldoorns Duizeligheidscentrum, Gelre Ziekenhuizen, Apeldoorn
- Maastrichts Universitair Medisch Centrum, Maastricht
- Haga-ziekenhuis, Den Haag
- Medisch Spectrum Twente, Enschede
- Rotterdam Duizeligheids Centrum, Rotterdam

Eligibility criteria	10	**Inclusion and exclusion criteria for participants. If applicable, eligibility criteria for study centres and individuals who will perform the interventions (eg, surgeons, psychotherapists)**

Inclusion criteria:

- Unilateral, definite MD according to the diagnostic criteria derived from the American Academy Otolaryngology Head and Neck Surgery, Classification- Page 4 of 6 Committee of the Bárány Society, European Academy of Otology and Neurotology and International Classification of Vestibular Disorders
- Adult patient (≥ 18 years), patient hospitalized
- ≥ 4 vertigo attacks over the last 6 months.
- Willing to adhere to daily Dizzy quest app questionnaires and the follow-up assessments..

Exclusion criteria:

- Bilateral MD
- Severe disability (e.g. neurological, orthopaedic, cardiovascular) or serious concurrent illness that might interfere with treatment or follow-up.
- Active additional neuro-otologic disorders that may mimic MD (e.g. vestibular migraine, recurrent vestibulopathy, phobic postural vertigo, vertebro-basilar TIAs, acoustic neuroma).
- Otitis media with effusion based on tympanogram results.
- History of intratympanic injections with corticosteroid less than 6 months ago.
- History of intratympanic injections with gentamicin or ear surgery for treating MD.
- Pregnant women and nursing women

Interventions	11a	**Interventions for each group with sufficient detail to allow replication, including how and when they will be administered**

Eligible patients will be randomised in equal proportion between the Methylprednisolone (62.5 mg/ml) and the Placebo (sodium chloride 0.9%). group. Intratympanic injections will be administered two times at timepoint 0 and after two weeks.

Name and description of investigational product(s):

*M*ethylprednisolone sodium succinate 62.5 mg/ml (Solu-Medrol in Act-O-Vial).

Generic name methylprednisolone sodium succinate. *Manufacture a.o.:* Pfizer B.V.; Rivium Westlaan 142 2909 LD Capelle a/d Ijssel, The Netherlands

Name and description of Placebo:

Placebo: Saline, Sodiumchloride (NaCl) 0,9% saline for injection

11b **Criteria for discontinuing or modifying allocated interventions for a given trial participant (eg, drug dose change in response to harms, participant request, or improving/worsening disease)**

Subjects can leave the study at any time for any reason if they wish to do so without any consequences. The investigator can decide to withdraw subjects from the study for urgent medical reasons. We do not anticipate any major events arising from the study intervention because the trail medicine is already widely used and the study is classified as a low risk profile study.

11c **Strategies to improve adherence to intervention protocols, and any procedures for monitoring adherence (eg, drug tablet return, laboratory tests)**

Patients cannot fail to take the medication because the intervention is an injection administered by the physician at timepoint zero and after two weeks. However, patients are obliged to complete short questionnaires on the DizzyQuest app on a daily basis. We will obtain a weekly output of the patients compliance to the app, which will aid in patient monitoring. To avoid data loss, we will contact patients who fail to report through the application and ask them to complete the questionnaires. We expect no problems assessing the other outcomes because they will be monitored throughout clinical or telephonic appointments.

11d **Relevant concomitant care and interventions that are permitted or prohibited during the trial**

Prohibited medication:

- The administration of antihistaminic drugs including betahistine is prohibited due to the risk of drug competition. The true effect of methylprednisolone could therefore be over- or underestimated

Specific permitted medication:

- In case subjects continue to suffer from vertigo attacks in a high frequency, co-interventions can be offered, in either in the placebo,- or the intervention group. Co interventions include: intratympanic injections of gentamicin, dexamethasone, methylprednisolone or triamcinolone.

- Subjects are allowed to use metoclopramide in the acute phase of vertigo

Outcomes 12 **Primary, secondary, and other outcomes, including the specific measurement variable (eg, systolic blood pressure), analysis metric (eg, change from baseline, final value, time to event), method of aggregation (eg, median, proportion), and time point for each outcome. Explanation of the clinical relevance of chosen efficacy and harm outcomes is strongly recommended**

**Primary objective(s)**	**Endpoint for the primary objective(s)**	**Analysis metric primary endpoint**	**Timepoints for primary objectives:**
To determine whether intratympanic injection with 62.5 mg/ml methylprednisolone is superior to placebo in reducing the frequency of vertigo attacks in patients diagnosed with unilateral MD during one year of follow-up.	Complete or substantial control of vertigo	Measured daily with the DizzyQuest app.<br>Categorized in class of vertigo (A-D)	Daily
**Secondary objective(s), if applicable**	**Endpoint(s) for secondary objective(s), if**	**Analysis metric**<br>**Secondary endpoint**	**Timepoints for secondary objectives:**
Use of co-interventions	Use of intratympanic injections with either gentamicin, methylprednisolone dexamethasone or triamcinolone	Frequency co-intervention used	Asked at 3, 6, 9 and 12 months
Use of Escape medication	Use of metoclopramide	Frequency escape medication used	Asked at 3, 6, 9 and 12 months
Hearing	Pure tone audiometry, word recognition score.	A decrease of ≥ 10 dB or a change in word recognition score of ≥ 15% points is considered clinically significant.	Measured at baseline, after 6 and 12 months
Change in quality of life on dizziness related quality of life	Questionnaires: Dizziness handicap inventory (DHI), Functional level scale (FLS)	DHI: handicap levels: Mild handicap = 16-34 Points; Moderate handicap = 36-52 Points and Severe handicap = 54+ Points.<br>Improved, unchanged of worsened of level.<br><br>FLS: Improvement (≥ 1 point decrease), unchanged or worsened (≤ 1 point increase)	Measured at baseline, after 6 and 12 months
Change in quality of life on tinnitus	Tinnitus handicap inventory (TFI)	Improvement (≥ 1 point decrease), unchanged or worsened (≤ 1 point increase)	Measured at baseline, after 6 and 12 months
Change in quality of life in general	EQ-5D and EQ-VAS scores	Mean value, standard deviation or, if the data is skewed, the median values and the 25th and 75th percentiles.	Measured at baseline, after 6 and 12 months
Adverse events	SAE, SUSARs	Frequency SAE/SUSAR	Reported when it occurs
Incremental cost differences	Cost-effectiveness and cost-utility analysis. Medical Consumption Questionnaire (iMCQ) and IMTA productivity Cost Questionnaire (iPCQ)	Cost-effectiveness analysis (costs per prevented vertigo attack), and a cost-utility analysis (costs per QALY, calculated from the EQ-5D and EQ-VAS	Measured at baseline, after 6 and 12 months

13 **Time schedule of enrolment, interventions (including any run-ins and washouts), assessments, and visits for participants. A schematic diagram is highly recommended (see Figure**

Participant timeline

Assessments	Screening Eligibility<br>Clinic<br>(-8 days till day -1)	Treatment visit 1<br>Clinic<br>Baseline (± 5 days)	Treatment visit 1<br>Clinic<br>2 weeks (± 5 days)	Follow-up phase<br>Telephone<br>3 months (± 2 weeks)	Follow-up phase<br>Clinic<br>6 months (± 2 weeks)	Follow-up phase<br>Telephone<br>9 months (± 2 weeks)	End of trial<br>Clinic<br>12 months (± 2 weeks)
Explanation study participation to subject	X						
Screening procedure	X						
Sign Informed Consent	X						
Inclusion/exclusion criteria	X						
Medical History	X						
Baseline parameters (sex, age, onset age of MD etc.)	X						
Concomitant medication	X			X	X	X	X
Pure Tone (PTA) and Speech Discrimination Score (SDS)	X[1,3]				X		X
Vestibular tests (caloric testing and vHIT)	X[3]						
MRI – petrous bone	X[3]						
Functional Level Scale (FLS)	X				X[2]		X
Dizziness Handicap Inventory (DHI)	X				X[2]		X
Tinnitus Functional Index (TFI)	X				X[2]		X
Generic quality of life questionnaires (EQ-5D, EQ-VAS)	X				X[2]		X
IMTA Medical Consumption Questionnaire (iMCQ) and IMTA productivity Cost Questionnaire (iPCQ)	X				X[2]		X
Randomisation	X						
Methylprednisolone or placebo		x	x				X
(S)AEs		x	x	X	X	X	X
Co intervention: injection with either methylprednisolone, dexamethasone, triamcinolone or gentamicine				Daily			
Dizzy quest app	Daily						

Sample size calculations	14	**Estimated number of participants needed to achieve study objectives and how it was determined, including clinical and statistical assumptions supporting any sample size**<br>The sample size calculation is based on recommendations as summarized in the Clinical Practice Guideline by Bassura et al [1]. The absolute effect on vertigo control class is expected to be 87.5% in the methylprednisolone group versus 67.5% in the placebo group. In previous studies similar high percentages of control of vertigo were seen when placebo or sham surgery was compared to an intervention. Sample size calculation was executed with the software available from DSS Research Tools (https://www.dssresearch.com/KnowledgeCenter/toolkitcalculators/statisticalpowercalculators.aspx analyses performed with the expected 20% difference in absolute effect on vertigo and two-tailed testing). With a statistical power of 80% and a Type 1 error of 5% 67 patients per group are required. With an estimated 10% loss-to-follow-up, we aim to include 74 patients in each group.
Recruitment	15	**Strategies for achieving adequate participant enrolment to reach target sample size.**<br>To achieve an adequate sample size, six centres in the Netherlands, with a specific department specialized in vertigo, are participating. In these centres, there is one ENT-surgeon who is in charge of the trial and recruits patients from their outpatient clinic. Presentations to inform medical professionals and to promote the study are held in these centres. When a patient is eligible, the coordinating investigator or research nurse will arrange for all of the study's requirements in order to keep the burden for the ENT-surgeons on the trial sites as low as possible. The coordinating investigator will be full-time involved in the PREDMEN-trial.<br>Additionally, the trial will be promoted in hospitals throughout the Netherlands, national congresses, informative websites on MD, and online patient communities.

Methods: Assignment of interventions (for controlled trials)

Allocation:

Sequence generation	16a	**Method of generating the allocation sequence (eg, computer- generated random numbers), and list of any factors for stratification. To reduce predictability of a random sequence, details of any planned restriction (eg, blocking) should be provided in a separate document that is unavailable to those who enrol participants or assign Interventions**<br>Subjects will be randomly assigned to either methylprednisolone or placebo with a 1:1 allocation as per computer-generated random sequence of block sizes of 2 and 4 subjects, stratified by site generated by Castor EDC ® SLL certified
Allocation concealment mechanism	16b	**Mechanism of implementing the allocation sequence (eg, central telephone; sequentially numbered, opaque, sealed envelopes), describing any steps to conceal the sequence until interventions are assigned**<br>Participants will be randomised using Castor EDC ® SLL certified, which is an online data management program. This program will send the randomisation code for a specific trail

participant to the pharmacy who is preparing the medication. In this way allocation concealment will be ensured to all concealed parties (physicians, research nurses, patients, data manager, statistician etc). Trial subjects, treating physicians and outcome assessors will be blinded throughout the entire study.

Implementation	16c	**Who will generate the allocation sequence, who will enroll participants, and who will assign participants to interventions**<br><br>All patients who give their consent and meet the inclusion criteria will be randomised. The ENT-surgeon or Research nurse in charge of recruitment will request randomization via mail, castorEDC, or phone to the coordinating investigator. Only the coordinating investigator can randomise patients. An automated email to the pharmacy with the randomization code will then be sent. On the day of allocation, the trial pharmacist will prepare the blinded trial medication (either methylprednisolone or placebo) and give it to the ENT surgeon for injection.
Blinding (masking)	17a	**Who will be blinded after assignment to interventions (eg, trial participants, care providers, outcome assessors, data analysts), and how**<br><br>Any individual involved in the trail, except for the pharmacy staff, will be blinded. This implies that all trial subjects, treatment physicians/nurses, outcome assessors and statisticians, will remain blinded until the trial's completion.
	17b	**If blinded, circumstances under which unblinding is permissible, and procedure for revealing a participant's allocated intervention during the trial**<br><br>Emergency unblinding may occur in the following situations: in case of a medical emergency where knowledge of the blinded treatment is necessary, for the treatment of (serious) adverse event, in the event of a SUSAR (Suspected Unexpected Serious Adverse Reaction) needing expedited reporting. The investigator must document the action taken and promptly notify the sponsor. Code breaks should only occur in exceptional circumstances as mentioned above and if it is absolutely essential for further management of the participant.

Methods: Data collection, management, and analysis

Data collection methods	18a	**Plans for assessment and collection of outcome, baseline, and other trial data, including any related processes to promote data quality (eg, duplicate measurements, training of assessors) and a description of study instruments (eg, questionnaires, laboratory tests) along with their reliability and validity, if known. Reference to where data collection forms can be found, if not in the protocol.**<br><br>Primary/Secondary outcomes: After a written informed consent is retrieved, subjects will visit the study site for the treatment with methylprednisolone/placebo at day 1 and at day 15 (with a visit window of 5 days), during a follow-up visit at 6 months, and during the end of trial visit at 12 months after first study treatment. Telephone contacts will take place at 3 and 9 monthspost-study treatment to discuss DizzyQuest app compliance and the occurrence of adverse events and use of concomitant medication. Tables presented in *question 12* and *13* display the outcome measurements collected.<br><br>Training + certification: Each individual involved in the trial must be in possession of a good

clinical practice (GCP) or BROK-certificate. In order to train all trail personnel, presentations at each trial site were held during the initiation visitation to discuss all study requirements and standard operating procedures.

18b **Plans to promote participant retention and complete follow-up, including list of any outcome data to be collected for participants who discontinue or deviate from intervention protocols.**

Patients will be closely monitored during the entire trial. Every Monday we will receive an output of patients' DizzyQuest usage, which will help us monitor compliance of patients. To avoid data loss, we will get in contact with patients who fail to report through the application and ask them to complete the questionnaires. Additionally, patients will have in-depth conversations and be questioned about their experiences during the trial at every follow-up visit, in which we can trace and solve potential obstacles.

Data management 19 **Plans for data entry, coding, security, and storage, including any related processes to promote data quality (eg, double data entry; range checks for data values). Reference to where details of data management procedures can be found, if not in the protocol**

The handling of personal data complies with the Dutch Personal Data Protection Act (in Dutch: De Wet Algemene Verodering Gegevensbescherming, AVG). All data collected for the trail, including but not limited to demographic data, audiological questionnaires, data from the DizzyQuest app will be entered in a ISO 9001 and ISO 27001:2005 certified Castor® EDC (electronic CRF). Personal and medical data that needs to be collected for the trail will be extracted from electronic medical records. We will use an unambiguous subject identification code that allows identification of all the data reported for each subject. The subject code will be documented on the subject identification list which will be filed in the ISF. There will be a subject identification list per participating site. The subject identification list, and any other data/documents containing personal identifiable information, of the participating site will remain at the site and will not be shared with the sponsor (LUMC) or any other participating site. Only members of the study team, who will be documented on the site signature and delegation log per site, will have access to the study data.

Analysed and processed data will be kept in a secured folder on the ENT department network drive of the LUMC, with restricted access to only the members of the study team. Every night backups are saved by the hospital IT automatically. The digital trial master file will be saved in PaNaMa RMS the research management system of the LUMC. Any hard copy documents will be stored in a locked cabinet at the ENT department of the LUMC. The (digital) investigator site files of the participating sites will be stored according to local procedures following the applicable regulations. The blinding is safeguarded by the pharmacies of all participating trial sites. If the study will not continue, all essential documents will be maintained for at least 2 years after formal discontinuation. All data will be stored for 25 years after the last subject has had the last study visit.

Statistical methods 20a **Statistical methods for analysing primary and secondary outcomes. Reference to where other details of the statistical analysis plan can be found, if not in the protocol**

Ordinal regression using mixed model analysis will be used to analyse the primary outcome

(i.e., class of vertigo). In addition, generalized estimating equation (GEE) analysis of the actual vertigo attacks recorded using the DizzyQuest app will be used to estimate the incidence rate ratio (IRR) for comparison between the methylprednisolone and placebo groups.

Mixed model analysis will be used to analyse differences in the questionnaire scores (DHI, TFI, FLS, eQ-5D/VAS, iMCQ, iPCQ) between the two groups. Logistic regression analysis will be used to analyse the remaining secondary outcomes (incidence of escape interventions, hearing loss, and adverse events).

In order to evaluate the average costs and outcomes between the methylprednisolone and placebo groups for the cost-effectiveness analysis, intention-to-treat and net-benefit analysis will be used. For all statistical analysis, multiple imputation to adjust for missing data will all be used. QALYs will be calculated using the Dutch tariff for the EuroQoL EQ-5D-5L and as sensitivity analysis the visual analogue scale valuing health, with power-transformation

20b **Methods for any additional analyses (eg, subgroup and adjusted analyses)**

Subgroup analyses will be performed with regard to sex, duration of the disease and the type of MD. Two sensitivity analyses will be carried out in addition to the intention to treat analysis: a per protocol analysis in which patients who received additional co-interventions to achieve vertigo control are excluded; and an as-treated analysis in which participants who received additional co-interventions are analysed.

20c **Definition of analysis population relating to protocol non-adherence (eg, as randomised analysis), and any statistical methods to handle missing data (eg, multiple imputation)**

Missing, unused and spurious data values will be coded as ‘888’; ambiguous values (e.g. if two or more boxes are ticked for a single dimension) will be treated as missing data. multiple imputations will be used to account for missing data.

Methods: Monitoring

Data monitoring

21a **Composition of data monitoring committee (DMC); summary of its role and reporting structure; statement of whether it is independent from the sponsor and competing interests; and reference to where further details about its charter can be found, if not in the protocol.**
Alternatively, an explanation of why a DMC is not needed

Since the intervention is categorized a low risk profile study no Data Safety Monitor Board (DSMB) or Data Monitoring Committee (DMC) is required and patient safety and treatment efficacy will we be performed by the independent expert.

21b **Description of any interim analyses and stopping guidelines, including who will have access to these interim results and make the final decision to terminate the trial.**

Interim analysis will be performed on the primary endpoint when 50% of the patients have been randomized and completed a follow-up of 6 months, where comparability of baseline characteristics will be assessed. In this analyses, differences in vertigo control between the two study arms should not be greater than 45%. In addition, if the difference

in vertigo control reveals to be clinically significant (i.e. >20%), but ≤ 20% of the participants in methylprednisolone reach vertigo control, the study will be terminated because of convincing effect of the treatment in the intervention arm.

Harms | 22 | **Plans for collecting, assessing, reporting, and managing solicited and spontaneously reported adverse events and other unintended effects of trial interventions or trial conduct**

Patients will be informed that Adverse Events (AE), Serious Adverse Events (SAE) and Suspected Unexpected Serious Adverse Reactions (SUSARs) must be reported as soon as possible to their ENT-surgeon or research nurse. Additional queries are made at 3, 6, 9, and 12 months to ensure that they did not fail to report occurrences. These events will be registered throughout the trial in Castor EDC.

Each SAE must be reported to the sponsor site within 24 hours after the physicians' knowledge. A SUSAR must be reported depending on the seriousness of the reaction and will be as follows:

- In the case of fatal or life-threatening SUSARs, not later than **7 days** after the sponsor became aware of the reaction
- In the case of non-fatal or non-life-threatening SUSARs, not later than **15 days** after the sponsor became aware of the reaction
- In the case of a SUSARs which was initially considered to be non-fatal or nonlife threatening but which turns out to be fatal or life-threatening, not later than **7 days** after the sponsor became aware of the reaction being fatal or life-threatening

The sponsor site will report the SAEs or SUSAR through the web portal of CTIS that approved the protocol, within 15 days after the sponsor has first knowledge of the serious adverse event.

Auditing | 23 | **Frequency and procedures for auditing trial conduct, if any, and whether the process will be independent from investigators and the sponsor**

Monitoring in all sites in the Netherlands will be executed by one assigned monitor of the LUMC according to the monitor plan. The monitor will visit each centre yearly. The visitation frequency can be altered based on the amount of inclusions. During the visitation the monitor will verify:

- Source data verification
- In,- and exclusion criteria
- Informed consent
- Control of the Trial Master File
- SAE's/SUSARS
- Trial procedures
- Product accountability
- Visitation of the pharmacy

The principal investigator of a participating centre receives a summary of the findings after each monitoring visit. The monitor report includes: a summary of reviewed trial data; a general description of the quality; a summary of key findings / facts, deviations and deficiencies; an overview of measures and recommendations to ensure compliance with the protocol; an "overall" conclusion.

Ethics and dissemination

Research ethics approval	24	**Plans for seeking research ethics committee/institutional review board (REC/IRB) approval**<br>The PREDMEN trial was submitted via the Clinical Trial Information System (CTIS), with CTIS number: *2023-503340-13-00*, reviewed by the Medical Review Research Ethics Committee Leiden The Hague Delft (MREC LDD), and authorized for execution in the Netherlands under the European Clinical Trial Regulation (ECTR), with ClinicalTrials.gov ID: *NCT05851508.* Additionally, the institutional research board of each participating centre individually reviewed and approved the study. The study is conducted in accordance with the principles outlined in the Declaration of Helsinki (October 2013), the Medical Research Involving Human Subjects Act (WMO, 26 February 1998), the International Conference on Harmonization Good Clinical Practice (ICH GCP, November 2016) guidelines, and any other applicable guidelines, regulations, and Acts.
Protocol amendments	25	**Plans for communicating important protocol modifications (eg, changes to eligibility criteria, outcomes, analyses) to relevant parties (eg, investigators, REC/IRBs, trial participants, trial registries, journals, regulators)**<br>Any modifications to the protocol which may impact on the conduct of the study, affecting the patients, study procedures, or significant administrative aspects will require an official amendment to the protocol. This amendment must be approved in the clinical trials information system.<br>Minor corrections to the protocol, which have no impact on the study, will be discussed with the sponsor's research team, and these minor corrections will be reported to the clinial trials information system if a major revision is submitted.
Consent or assent	26a	**Who will obtain informed consent or assent from potential trial participants or authorised surrogates, and how (see Item 32)**<br>Patients will be introduced to the trail and given a patient information letter by trained research nurses, ENT-surgeons, or the coordinating investigator. After a few days, research nurses or the coordinating investigator will have an informed consent conversation with patients and answer their questions. When the patient agrees to participate in the trial, the research nurse, involved ENT-surgeon or coordinating investigator will obtain a written informed consent.
	26b	**Additional consent provisions for collection and use of participant data and biological specimens in ancillary studies, if applicable**<br>Not applicable
Confidentiality	27	**How personal information about potential and enrolled participants will be collected, shared, and maintained in order to protect confidentiality before, during, and after the trial.**<br>Source documents for this study will include hospital records and procedure reports and

data collection forms. These documents will be used to enter data on the (e)CRFs. Data reported on the (e)CRF that are derived from source documents must be consistent with the source documents or the discrepancies must be explained. On all study-specific documents other than the signed consent, the subject will be referred to by the study subject identification code.

Each study site has its own investigator site file, where study specific information is stored. This ISF will be secured with password-protected access systems, available to trial personnel only. All other participant information such as signed consent forms, will be stored in locked file cabinets in areas with limited access on the trial site.

DizzyQuest app: All data handling will be documented in a processing agreement between the LUMC and the company that created the DizzyQuest App (Psymate). Until a reliable internet connection is established, the user's smartphone will store the DizzyQuest app's data. The smartphone stores data that is encrypted and inaccessible to outside parties. Data will be sent to the Smart eHealth servers in Belgium when the smartphone connects to the internet. All data will be automatically removed from the smartphone after the data transfer.

Declaration of interests	28	**Financial and other competing interests for principal investigators for the overall trial and each study site**<br>None
Access to data	29	**Statement of who will have access to the final trial dataset, and disclosure of contractual agreements that limit such access for investigators**<br>Only the coordinating investigators and PI of the sponsor site will have access to the entire data set. Other principal investigators will have direct access to their own site’s dataset and will have access to a cleaned data set of all sites on request. To ensure confidentiality, any identifying participant information will be removed from data distributed to project team members.
Ancillary and post-trial care	30	**Provisions, if any, for ancillary and post-trial care, and for compensation to those who suffer harm from trial participation**<br>The study has been classified as a low risk profile study. Therefore we do not anticipate any harm from trial participation. When the trial has completed after one year, the patient will be returned to the care of his personal ENT-surgeon.<br>However, when any harm from trial participation occurs, the sponsor has an insurance that is in accordance with the legal requirements in the Netherlands (Article 7 WMO, under 1). This insurance provides cover for damage to research subjects through injury or death caused by the study. The insurance applies to the damage that becomes apparent during the study or within 4 years after the end of the study.

Dissemination policy 31a **Plans for investigators and sponsor to communicate trial results to participants, healthcare professionals, the public, and other relevant groups (eg, via publication, reporting in results databases, or other data sharing arrangements), including any publication restrictions .**

After completion of the trial, all patients who wish to know to which study arm they were allocated, will be informed.

Within one year from the end of a clinical trial in all Member States concerned, the sponsor will submit to the EU database CTIS a summary of the results of the clinical trial. The content of the summary of the results is set out in CTR Annex IV. It shall be accompanied by a summary written in a manner that is understandable to laypersons. The content of the summary is set out in CTR Annex V.

Furthermore, abstracts, papers and oral presentations will be submitted to several symposia and scientific papers. There will be two classes of reports of the PREDMEN trial:

A. Reports of the major outcomes of the PREDMEN trial.
B. Reports addressing in detail on aspect of the PREDMEN trial

Each paper, abstract, or oral presentation will be reviewed and approved by the sponsor's research team.

31b **Authorship eligibility guidelines and any intended use of professional writers**

Researchers who have been involved from the beginning and are certain to receive authorship will be listed as below:

Abstracts/papers/oral presentation: M.M.E. Boreel MD, B.F. van Esch, MD, PhD, B.M. Mol, MSc PhD, prof. P.P.G. van Benthem, MD, PhD, prof. T.D. Bruintjes, MD, PhD.

These arrangements for publication are based on the following merits: M.M.E. Boreel is the PhD student and is working full time on the research. B.F. van Esch is the principal investigator, B.M. Mol is head of research support, P.P.G. van Benthem is professor and the head of the Otorhinolaryngology Department and. T.D. Bruintjes is Professor Otorhinolaryngology, in particular Ménière's disease, and project leader. Other authors of participating centres will be added to the manuscript according to their participation and number of subjects they have included.

31c **Plans, if any, for granting public access to the full protocol, participant- level dataset, and statistical code**

In a period of no more than 2 years after the collection of the 1-year post randomization interviews and diaries, we will deliver a completely deidentified data set to an appropriate archive for sharing purposes. In due course, consultations will be held with the data protection officer regarding the preparation of this dataset. So that it complies with Dutch and local (LUMC) laws and regulations.

Appendices

Informed consent materials	32	**Model consent form and other related documentation given to participants and authorised surrogates**
		The patient information letter and informed consent form is included in the appendix.
Biological specimens	33	**Plans for collection, laboratory evaluation, and storage of biological specimens for genetic or molecular analysis in the current trial and for future use in ancillary studies, if applicable**
		Not applicable

*It is strongly recommended that this checklist be read in conjunction with the SPIRIT 2013 Explanation & Elaboration for important clarification on the items. Amendments to the protocol should be tracked and dated. The SPIRIT checklist is copyrighted by the SPIRIT Group under the Creative Commons "Attribution-NonCommercial-NoDerivs 3.0 Unported" license.

References:

1. Basura, G.J., et al., *Clinical Practice Guideline: Ménière's Disease.* Otolaryngol Head Neck Surg, 2020. **162**(2_suppl): p. S1-s55.
2. Hamid, M. and D. Trune, *Issues, indications, and controversies regarding intratympanic steroid perfusion.* Curr Opin Otolaryngol Head Neck Surg, 2008. **16**(5): p. 434-40.
3. Jiang, M., Z. Zhang, and C. Zhao, *What is the efficacy of gentamicin on the incidence of vertigo attacks and hearing in patients with Meniere's disease compared with steroids? A meta-analysis.* Journal of Neurology, 2021. **268**(10): p. 3717-3727.
4. Patel, M., et al., *Intratympanic methylprednisolone versus gentamicin in patients with unilateral Ménière's disease: a randomised, double-blind, comparative effectiveness trial.* Lancet, 2016. **388**(10061): p. 2753-2762.
5. Webster, K.E., et al., *Intratympanic corticosteroids for Ménière's disease.* Cochrane Database Syst Rev, 2023. **2**(2): p. Cd015245.
6. Salt, A.N. and S.K. Plontke, *Pharmacokinetic principles in the inner ear: Influence of drug properties on intratympanic applications.* Hear Res, 2018. **368**: p. 28-40.
7. Parnes, L.S., A.H. Sun, and D.J. Freeman, *Corticosteroid pharmacokinetics in the inner ear fluids: an animal study followed by clinical application.* Laryngoscope, 1999. **109**(7 Pt 2): p. 1-17.
8. Cao, Z., et al., *Different medications for the treatment of Ménière's disease by intratympanic injection: A systematic review and network meta-analysis.* Clin Otolaryngol, 2019. **44**(4): p. 619-627.
9. Salt, A.N. and S.K. Plontke, *Principles of local drug delivery to the inner ear.* Audiol Neurootol, 2009. **14**(6): p. 350-60.
