# Supplementary material for: The effectiveness of intratympanic injections with methylPREDnisolon versus placebo in the treatment of vertigo attacks in MENière’s disease (PREDMEN trial): a study protocol for a phase-3 multicentre, double-blinded, randomised, placebo-controlled trial
Source: BMJ Open. 2024 Aug 29;14(8):e076872. doi: 10.1136/bmjopen-2023-076872 (PMC11367374; doi:10.1136/bmjopen-2023-076872)
Supplement: Supplementary file 3 [file bmjopen-2023-076872.reviewer_comments.pdf]

PEER REVIEW HISTORY

BMJ Open publishes all reviews undertaken for accepted manuscripts. Reviewers are asked to complete a checklist review form and are provided with free text boxes to elaborate on their assessment. These free text comments are reproduced below.

ARTICLE DETAILS

**TITLE (PROVISIONAL)**	The effectiveness of intratympanic injections with methylPREDnisolon versus placebo in the treatment of vertigo attacks in MENière's disease (PREDMEN trial): a study protocol for a phase-3 multicentre, double-blinded, randomized, placebo-controlled trial
**AUTHORS**	Boreel, Maud; van Esch, Babette; Schermer, Tjard; Mol, Berber M.; van Benthem, Peter Paul; Bruintjes, Tjasse D.

VERSION 1 - REVIEW

**REVIEWER NAME**	***Jose A Lopez-Escamez***
**REVIEWER AFFILIATION**	The University of Sydney Faculty of Medicine and Health, School of Medical Sciences
**REVIEWER CONFLICT OF INTEREST**	None
**DATE REVIEW RETURNED**	17-Jul-2023

**GENERAL COMMENTS**	This study is a description of the PREDMEN trial protocol, a RCT planned to include 148 patients with unilateral (Meniere's disease) MD in a multicenter study in the Netherlands.<br>Patients will be randomly assigned to either the methylprednisolone, or the placebo<br>groups. The intervention is planned by 2 injections, one at baseline and one after two weeks. Follow-up assessments will be done at 3, 6, 9 and 12 months. The primary outcome will be the frequency of vertigo attacks that will be evaluated in an app.<br><br>I have some concerns about the study design, the ethical approval, and the statement of the clinical researchers about the difficulties to define endophenotypes in MD.<br><br>1. There is evidence to support that some patients with MD may benefit from steroids according to observational (repeated response in the same patient) and clinical trial studies. For this reason, a crossover design with wash-out period will be more appropriate to give all the participants the same opportunity to be treated. This reviewer does not consider acceptable to use a placebo in patients with a confirmed diagnosis of MD and 6 attacks of vertigo (as stated in the inclusion criteria).<br><br>2. The main limitation of the RCTs that have been performed in the past have been the poor phenotyping of the participants or the "broad selection criteria" including patients with MD of any type (MD+migraine, familial MD, autoimmune MD, autoinflammatory MD). This study repeats the same biases that previous RCT and does not exclude patients with migraine or familial history of vertigo.

Current practice for RCT is the use of biomarkers as inclusion criteria or as outcome measure. The authors have not considered to include individuals with high levels of IgE, IL1B, TNFa, IL6 or IL4. These markers have been reported associated with endotypes of MD and associated with inflammation, allergy or autoimmunity.
The inclusion criteria are too broad and any subtype of patient with MD is planned to be included. If the researchers want to study all the MD endotypes, they will need a larger sample size. Alternatively, they could select the subgroup of patients with immune disorder (autoimmune or autoinflammation) and probably it will be expected a positive response.
Exclusion criteria should include: a) presence of migraine (to exclude MD type 4) and familial history of vertigo or sensorineural hearing loss (to exclude familial MD type 3, which it is associated with multiallelic recessive or monoallelic dominant inheritance). Patients with MD type 3 or 4 are unlikely to respond to steroids.
With the current design, we could anticipate that placebo and treatment groups will no show significant differences.

Essential references to define clinical or molecular subgroups in MD are not included and the researchers should seriously reconsider these studies to improve the study protocol. They should select at least one/two biomarkers and use it as outcome measure.

References

Flook M, Frejo L, Gallego-Martinez A, Martin-Sanz E, Rossi-Izquierdo M, Amor-Dorado JC, Soto-Varela A, Santos-Perez S, Batuecas-Caletrio A, Espinosa-Sanchez JM, Pérez-Carpena P, Martinez-Martinez M, Aran I, Lopez-Escamez JA. Differential Proinflammatory Signature in Vestibular Migraine and Meniere Disease. Front Immunol. 2019 Jun 4;10:1229. doi: 10.3389/fimmu.2019.01229. PMID: 31214186; PMCID: PMC6558181.

Flook M, Escalera-Balsera A, Rybakowska P, Frejo L, Batuecas-Caletrio A, Amor-Dorado JC, Soto-Varela A, Alarcón-Riquelme M, Lopez-Escamez JA. Single-cell immune profiling of Meniere Disease patients. Clin Immunol. 2023 Jul;252:109632. doi: 10.1016/j.clim.2023.109632. Epub 2023 May 11. PMID: 37178857.

Frejo L, Gallego-Martinez A, Requena T, Martin-Sanz E, Amor-Dorado JC, Soto-Varela A, Santos-Perez S, Espinosa-Sanchez JM, Batuecas-Caletrio A, Aran I, Fraile J, Rossi-Izquierdo M, Lopez-Escamez JA. Proinflammatory cytokines and response to molds in mononuclear cells of patients with Meniere disease. Sci Rep. 2018 Apr 13;8(1):5974. doi: 10.1038/s41598-018-23911-4. PMID: 29654306; PMCID: PMC5899176.

Frejo L, Martin-Sanz E, Teggi R, Trinidad G, Soto-Varela A, Santos-Perez S, Manrique R, Perez N, Aran I, Almeida-Branco MS, Batuecas-Caletrio A, Fraile J, Espinosa-Sanchez JM, Perez-Guillen V, Perez-Garrigues H, Oliva-Dominguez M, Aleman O, Benitez J, Perez P, Lopez-Escamez JA; Meniere's disease Consortium (MeDiC). Extended phenotype and clinical subgroups in unilateral Meniere disease: A cross-sectional study with cluster analysis. Clin Otolaryngol. 2017 Dec;42(6):1172-1180. doi: 10.1111/coa.12844. Epub 2017 Feb 26. PMID: 28166395.

	Lopez-Escamez JA, Vela J, Frejo L. Immune-Related Disorders Associated With Ménière's Disease: A Systematic Review and Meta-analysis. Otolaryngol Head Neck Surg. 2023 Jun 5. doi: 10.1002/ohn.386. Epub ahead of print. PMID: 37272729.<br><br>Moleon MD, Martinez-Gomez E, Flook M, Peralta-Leal A, Gallego JA, Sanchez-Gomez H, Montilla-Ibañez MA, Dominguez-Durán E, Soto-Varela A, Aran I, Frejo L, Lopez-Escamez JA. Clinical and Cytokine Profile in Patients with Early and Late Onset Meniere Disease. J Clin Med. 2021 Sep 7;10(18):4052. doi: 10.3390/jcm10184052. PMID: 34575163; PMCID: PMC8472422.<br><br>Zhang DG, Yu WQ, Liu JH, Kong LG, Zhang N, Song YD, Li XF, Fan ZM, Lyu YF, Li N, Wang HB. Serum/glucocorticoid-inducible kinase 1 deficiency induces NLRP3 inflammasome activation and autoinflammation of macrophages in a murine endolymphatic hydrops model. Nat Commun. 2023 Mar 6;14(1):1249. doi: 10.1038/s41467-023-36949-4. PMID: 36872329; PMCID: PMC9986248.<br><br>Zhang N, Lyu Y, Guo J, Liu J, Song Y, Fan Z, Li X, Li N, Zhang D, Wang H. Bidirectional Transport of IgE by CD23 in the Inner Ear of Patients with Meniere's Disease. J Immunol. 2022 Feb 15;208(4):827-838. doi: 10.4049/jimmunol.2100745. Epub 2022 Jan 19. PMID: 35046106; PMCID: PMC9012086.<br><br>Zou J, Zhao Z, Song X, Zhang G, Li H, Zhang Q, Pyykkö I. Elevated G-CSF, IL8, and HGF in patients with definite Meniere's disease may indicate the role of NET formation in triggering autoimmunity and autoinflammation. Sci Rep. 2022 Sep 29;12(1):16309. doi: 10.1038/s41598-022-20774-8. PMID: 36175465; PMCID: PMC9522806.

**REVIEWER NAME**	***Georgios Kontorinis***
**REVIEWER AFFILIATION**	University of Glasgow
**REVIEWER CONFLICT OF INTEREST**	None
**DATE REVIEW RETURNED**	19-Jul-2023

**GENERAL COMMENTS**	The authors present a study protocol, which is overall well set up and written.<br>My main concern are the study arms; given the current evidence, wouldn't it be worth but also scientifically more robust to include a third group of patients with either dexamethasone or even gentamicin? This would also answer questions not previously answered in direct comparison.<br>Another concern is the pain usually triggered by methylprednisolone itself; this introduces some bias to how really blind the study is.<br>Minor comments:<br>-line 65: the word unilateral can be debated as there are patients suffering from bilateral MD.<br>-line 72: the comment on effective treatment is also arguable: there is evidence to suggest the efficacy of certain treatment regimes; however, there are wide variances in practice and individualisation of treatment.<br>-line 154: the word potential should be eliminated

	-line 314: the word intratympanic should be eliminated; additionally 38% residual perforation following suh puncture sounds exaggeration<br><br>Have the authors considered documenting any potential or proven autoimmune backgro<br><br>Finally, in the methods, the authors should emphasise more on the injection technique as well as who will be performing these (what level of experience).

**REVIEWER NAME**	***Matthew Smith***
**REVIEWER AFFILIATION**	University of Cambridge
**REVIEWER CONFLICT OF INTEREST**	None
**DATE REVIEW RETURNED**	13-Oct-2023

**GENERAL COMMENTS**	This is a blinded randomised controlled trial relating to an important clinical issue. Overall the trial appears well-designed to answer the central clinical question. It will not be able to answer the question as to the optimal steroid choice dexamethasone Vs methylprednisolone, with a potential difference hypothesised in the introduction.<br><br>As a study protocol reporting is recommended based on SPIRIT guidelines. Although reporting is overall good, these have not been fully adhered to.<br><br>Inclusion criteria appear appropriate and adequately described<br><br>While the primary outcome measure has been described, the timing for the primary outcome is not clear in outcomes (6 or 12 months), or which time point sample size estimate applies to.<br><br>The sample size calculation gives predicted 10% loss to follow up, but this does not appear to be accounted for by increasing the sample size. The primary outcome is collected by app, and investigators should consider if self-directed data entry will be sufficient for analysis of 90% of participants - pilot data may be useful here. App data may be harder to ensure completeness when compared to conventional investigator-recorded data.<br><br>Additional minimisation criteria could be considered, as severity of baseline hearing loss and attack frequency may impact on the primary outcome. VHIT results to classify extent of vestibular hypo function at baseline may also help to balance groups. Minimisation for sex and age could also be considered.<br><br>Line 193: SAE data collection at 3 and 6 months appears late both in terms of accurate collection based on recall, and in detection and reporting of SAEs to the sponsor which would normally be quicker. It is noted that the intervention is low risk however and in use in routine clinical practice.<br><br>Regarding quality of life measures, the EQ-5D is poorer than the HUI-3 for detecting change in hearing-related disorders.

	Line 236 - it appears that crossover to IT methylpred will be allowed in the control arm. If this is widespread the planned ITT analysis will fail to detect a difference between arms. The primary analysis should be per protocol if significant cross over is expected, and PP as a sensitivity analysis. Investigators may wish to issue clear guidelines as to when crossover may be permitted.<br><br>The choice of outcomes and clinically important differences appears appropriate. Further details should be given on the frequencies tested for 'low-frequency hearing' and the speech test chosen.<br><br>Minor points:<br>Line 92: "Pharmacokinetic studies..." This sentence appears incomplete.<br>Few typos, e.g line 122 'trail'.

VERSION 1 – AUTHOR RESPONSE

Reviewer 1:

Comment 1: There is evidence to support that some patients with MD may benefit from steroids according to observational (repeated response in the same patient) and clinical trial studies. For this reason, a crossover design with wash-out period will be more appropriate to give all the participants the same opportunity to be treated.

Response 1: *We thank the reviewer for taking the time to read our manuscript and for giving insightful comments and feedback.*

The study design mentioned by the reviewer is indeed an interesting suggestion. However, the pharmacokinetic properties of methylprednisolone in the labyrinth are largely unknown. Therefore, no concrete information on the clinical wash-out period is present. A large sample size parallel placebo-RCT including methylprednisolone and placebo will provide us the information to estimate the true effect of this potentially useful drug in treating Menière's disease. We decided to adopt the much acclaimed study design by Patel et al. on intratympanic methylprednisolone versus gentamicin for Menière's disease [1].

Comment 2: The reviewer does not consider it acceptable to use a placebo in patients with a confirmed diagnosis of MD and 6 attacks of vertigo (as stated in the inclusion criteria).

Response 2: *We thank the reviewer for his critical comment. Until this day, an evidence-based treatment has not been established for MD. Although several interventions may be effective, to estimate the true size of the effect, the intervention needs to be compared to a placebo. As a result, the use of a placebo is a deliberate choice and is supported by the Medical Ethical Committee in our hospital. Apart from that, the protocol has been submitted to, and approved by the Clinical Trials Information System (CTIS) of the European Medicines Agency (EMA). Besides, our protocol states that when patients suffer from persistent vertigo in the follow-up period, co-interventions are provided in the form of intratympanic injections with either gentamicin or corticosteroid and any required treatment will therefore never be withheld.*

Comment 3: The main limitation of the RCTs that have been performed in the past has been the poor phenotyping of the participants or the "broad selection criteria" including patients with MD of any type (MD+migraine, familial MD, autoimmune MD, autoinflammatory MD). This study repeats the same biases that previous RCT and does not exclude patients with migraine or familial history of vertigo. Current practice for RCT is the use of biomarkers as inclusion criteria or as outcome measure. The authors have not considered to include individuals with high levels of IgE, IL1B, TNFa, IL6 or IL4. These markers have been reported associated with endotypes of MD and associated with

inflammation, allergy or autoimmunity. The inclusion criteria are too broad and any subtype of patient with MD 6laned to be included. If the researchers want to study all the MD endotypes, they will need a larger sample size. Alternatively, they could select the subgroup of patients with immune disorder (autoimmune or autoinflammation) and probably it will be expected a positive response.
Exclusion criteria should include: a) presence of migraine (to exclude MD type 4) and familial history of vertigo or sensorineural hearing loss (to exclude familial MD type 3, which it is associated with multiallelic recessive or monoallelic dominant inheritance). Patients with MD type 3 or 4 are unlikely to respond to steroids.
With the current design, we could anticipate that placebo and treatment groups will no show significant differences.

Response 3 *We thank the reviewer for highlighting this matter and we acknowledge the importance of detailed information on the different subtypes in MD. However, the subtyping for Menière's Disease mentioned by the reviewer is, as of yet, not used worldwide. To date, no therapeutic trial has been conducted that takes subtypes into account. Introducing subtypes in our trial would make comparison with previous trial results difficult. However, we will perform subgroup analyses for the different subtypes described by Frejo et al. [2].*

Although research shows that there seems to be a subset of MD patients with a persistent inflammatory status, solid evidence to support auto-inflammatory etiology in MD is still limited. Theoretically, it is plausible that these patients are likely to benefit from corticosteroid treatment, but this has not been proven yet. Furthermore, it is likely to assume that the proportion of these patients is more or less the same in any large group of MD patients participating in a clinical trial.

A practical obstacle to include the blood markers mentioned by the reviewer is that these are not standard laboratory tests that can be run in the trial sites included.

Unfortunately, incorporating a larger research population and performing additional clinical analysis as mentioned by the reviewer, would need much more time and money. Inclusion of MD patients in a clinical trial is a long lasting and time-consuming process (e.g. in the BEMED trial it took five years to include 221 Meniere patients in 14 centers) [3]. We think that the target number of patients to be included is already fairly high.

Comment 4: Essential references to define clinical or molecular subgroups in MD are not included and the researchers should seriously reconsider these studies to improve the study protocol. They should select at least one/two biomarkers and use it as outcome measure.

Response 4: *We thank the reviewer for this comment. We will perform a subgroup analysis using the subgroups proposed by Frejo et al. (2017). We will not perform a molecular subgroup analysis, because the biomarkers proposed are based on the results of a few studies from a single research group. Apart from that, we do not have the resources to fund additional clinical analysis.*

Reviewer 2:

Comment 1: The authors present a study protocol, which is overall well set up and written.
My main concern are the study arms; given the current evidence, wouldn't it be worth but also scientifically more robust to include a third group of patients with either dexamethasone or even gentamicin? This would also answer questions not previously answered in direct comparison.

Response 1: *We thank the reviewer for the valuable suggestion and the time put into reviewing this manuscript. Actually, we took into consideration the inclusion of a third study arm. However, we were concerned that the population size required would then not be feasible (see response 3, reviewer 1). This is why we chose to compare the highest methylprednisolone dose (also used by Patel et al. in their study) with placebo to investigate its potential clinical efficacy rather than the possibly existing dose-response relation [1].*

Comment 2: Another concern is the pain usually triggered by methylprednisolone itself; this introduces some bias to how really blind the study is.

Response 2: *This is a concern also addressed by Patel. et al in their study comparing methylprednisolone and gentamicin [1]. Fortunately, they found that no patient identified which drug they received on the basis of their side-effects. For the sake of completeness we added in our protocol that patients can, if they wish, take pain medication such as paracetamol beforehand to reduce possible pain.*

Minor comments:

Comment 4: line 65: the word unilateral can be debated as there are patients suffering from bilateral MD.

Response 4: *It is correct that patients can suffer from bilateral MD. We have eliminated the word unilateral.*

Comment 5: line 72: the comment on effective treatment is also arguable: there is evidence to suggest the efficacy of certain treatment regimens; however, there are wide variances in practice and individualisation of treatment.

Response 5: *In agreement with the reviewer, the statement could be somewhat more nuanced. This has been altered in the text (lines 72-73).*

Comment 6: line 154: the word potential should be eliminated

Response 6: *The word potential has been eliminated*

Comment 7: line 314: the word intratympanic should be eliminated; additionally 38% residual perforation following such puncture sounds exaggeration.

Response 7: *The word intratympanic has been eliminated. Furthermore, we found a more accurate reference on the chance of persistent membrane perforation after intratympanic injection and changed this in the manuscript (line 341).*

Comment 8: Have the authors considered documenting any potential or proven autoimmune background?

Response 8: *We have considered documenting autoimmune background. All patients will be asked about their medical history of auto-immune diseases. Additional questions were implemented in order to categorize participants into the various MD subtypes, proposed by Frejo et al [2]. Subsequently, a sub-analysis will be conducted based on these subtypes. This has been addressed more extensively in lines 187 – 189.*

Comment 9: Comment 9 Finally, in the methods, the authors should emphasize more on the injection technique as well as who will be performing these (what level of experience).

Response 9: *We thank the reviewer for pointing this out, we have described the method of injection more extensive in lines 191 – 194.*

Reviewer: 4

Comment 1: This is a blinded controlled trial relating to an important clinical issue. Overall the trial appears well-designed to answer the central clinical question. It will not be able to answer the question as to the optimal steroid choice dexamethasone Vs methylprednisolone, with a potential difference hypothesized in the introduction.

Response 1: *We thank the reviewer for taking the time to evaluate this manuscript and for providing critical feedback*.

We agree that the research question on the optimal choice of corticosteroids is an interesting one. However, pharmacokinetic research has shown that dexamethasone is not well-suited for inner ear therapy. Once within the inner ear dexamethasone is rapidly eliminated from the perilymph. Methylprednisolone seems to have properties more appropriate for intratympanic application (as demonstrated in a clinical trial by Patel et. al) [1, 4-6].

In addition, to answer this research question we would have to include a third treatment arm In line with the response number 3 on the first reviewer inclusion of MD patients in a clinical trial is a long lasting and time-consuming process (e.g. in the BEMED trial it took five years to include 221 Meniere patients in 14 centers) [3]. We think that the target number of patients to be included is already fairly high and we for see that inclusion of that many patients will not be realistic given the participating trial sites and the time period. (see response 3, reviewer 1).

Comment 2: As a study protocol reporting is recommended based on SPIRIT guidelines. Although reporting is overall good, these have not been fully adhered to. –

Response 2: *Thank you for addressing this point, the SPIRIT checklist will be provided as a supplementary file.*

Comment 3: While the primary outcome measure has been described, the timing for the primary outcome is not clear in outcomes (6 or 12 months), or which time point sample size estimate applies to.

Response 3: *We agree, and have explained in more detail how the primary outcome measures are being measured (lines 201-202).*

Comment 4: The sample size calculation gives a predicted 10% loss to follow-up, but this does not appear to be accounted for by increasing the sample size.

Response 4: *The 10 % is accounted for in the calculation, however, we did not explain how the calculation was made. In the text, it is now explained how the 10% loss to follow-up was calculated. (lines 170-172)*

Comment 5: The primary outcome is collected by the app, and investigators should consider if self-directed data entry will be sufficient for analysis of 90% of participants - pilot data may be useful here. App data may be harder to ensure completeness when compared to conventional investigator-recorded data.

Response 5: *This is an important point. Fortunately, colleagues from Maastricht University Medical Center (MUMC) and Haga-Hospital, who also use this app for research, see that the compliance for data completion in the DizzyQuest app is very high. Additionally, to overcome this problem we will receive an output of the patients who fill in the app every week. If patients are not consequent in reporting in the application we will contact them to ensure they will fill in the app to have as minimal loss of data as possible. Besides, by using a daily app where patients can report their vertigo attacks, we will avoid recall bias. However, the completeness of data can still be a challenge in this research.*

Comment 6 : Additional minimisation criteria could be considered, as severity of baseline hearing loss and attack frequency may impact on the primary outcome. VHIT results to classify extent of vestibular hypo function at baseline may also help to balance groups. Minimisation for sex and age could also be considered.

Response 6: *We thank the editor for its recommendation, the aim of applying minimisation is to minimize the imbalance between the number of patients in each treatment group over a number of factors. However, as the reviewer said, this would only be required if these factors affect the outcome measures.*

Because hearing loss in Meniere's disease fluctuates, categorizing people into hearing loss groups may misinterpret the outcomes, and therefore, we will not use minimisation based on hearing loss stage. However, we will perform subgroup analyses based on the stage of hearing loss. The

relationship between vertigo and the duration of the disease is quite complicated. Perez et al. showed that there is a relationship between the frequency of vertigo episodes and disease stage, although this correlation rapidly diminishes as the disease progresses [7]. The vHIT test result is an unreliable way to classify the extent of vestibular hypofunction, because in MD disease progression does not often result into an abnormal vHIT result [8]. Because the factors mentioned above do not show a clear correlation with the outcome measures, we chose not to use minimisation.

Since we will include 148 patients in our trial, we assume that both groups will be evenly distributed and minimisation for sex and age is not required. In addition, when stratifying on too many characteristics, the statistical power is compromised. During the analysis, the various characteristics mentioned by the reviewer will be examined and compared by group and, if necessary, corrected for.

Comment 7: Line 193: SAE data collection at 3 and 6 months appears late both in terms of accurate collection based on recall, and in detection and reporting of SAEs to the sponsor which would normally be quicker. It is noted that the intervention is low risk however and in use in routine clinical practice.

Response 7: *We agree with this point and it was not adequately explained in the manuscript. Patients will be informed that if they have an incident, they must notify their ENT-surgeon or research nurse so we can evaluate if this incident is an AE, SEA or SUSAR. In addition, investigators are required to report SAEs to the sponsor no later than 24 hours after the site becomes aware of the event. Additional queries are made at 3, 6, 9, and 12 months to ensure that they did not fail to report occurrences. This is elaborated in lines: 254-259*

Comment 8: Regarding quality of life measures, the EQ-5D is poorer than the HUI-3 for detecting change in hearing-related disorders.

Response 8: *We agree that the HUI-3 is more accurate in detecting changes in hearing loss. However, the EQ-5D is the questionnaire of recommendation aiming to perform a cost-effectiveness analysis.*

Comment 9: Line 236 - it appears that crossover to IT methylpred will be allowed in the control arm. If this is widespread the planned ITT analysis will fail to detect a difference between arms. The primary analysis should be per protocol if significant cross over is expected, and PP as a sensitivity analysis. Investigators may wish to issue clear guidelines as to when crossover may be permitted. –

Response 9: *Thank you for bringing this to our attention. In both arms, additional treatment is permitted. Patients are not de-blinded in this case. As a result, it is essential to keep track of the additional therapy so that we can compare the use of additional treatment in both groups. Therefore cross-over will not occur. This will be clarified in greater detail in the paper (lines 246-251)*

Comment 10: The choice of outcomes and clinically important differences appears appropriate. Further details should be given on the frequencies tested for 'low-frequency hearing' and the speech test chosen.

Response 10: *Agreed, this is more extensively clarified in the manuscript (lines 232-235).*

Minor points:

Comment 11: Line 92: "Pharmacokinetic studies..." This sentence appears incomplete.
Few typos, e.g line 122 'trail'.

Response 11: *Agreed, clarified sentence: Lines 93-95, agreed, changed typo's*

VERSION 2 – REVIEW

**REVIEWER NAME**	***Georgios Kontorinis***
**REVIEWER AFFILIATION**	University of Glasgow
**REVIEWER CONFLICT OF INTEREST**	None
**DATE REVIEW RETURNED**	21-Jan-2024

**GENERAL COMMENTS**	The authors have taken into account all my comments. One could argue the lack of a third arm but the logistics of such protocol change would make the study non feasible. As long as this is addressed during the study, then it should be adequate.
